# Plaque‐type trichoblastoma and its siblings: A (follicular tumour) family history

**DOI:** 10.1111/ddg.70255

**Published:** 2026-03-19

**Authors:** Ines Bertlich, Ferdinand Toberer, Tilman Schulz, Dmitry V. Kazakov, Wolfgang Hartschuh

**Affiliations:** ^1^ University Hospital Heidelberg, Department of Dermatology, INF 440 Heidelberg Germany; ^2^ Institute of Pathology Klinikum Bayreuth GmbH Bayreuth Germany; ^3^ IDP Institut für Dermatohistopathologie Pathologie Institut Enge Zürich Switzerland; ^4^ Hautarztpraxis Durani Heidelberg Germany

Dear Editors,

A 61‐year‐old female patient had noticed a plaque in her left nasolabial fold for two years, which had started to itch and to increase in size in the past six months. Clinical examination showed an ill‐circumscribed, skin‐colored plaque with coarse telangiectasia, measuring approx. 4 mm in diameter (Figure 1a–c). We performed optical coherence tomography (OCT). It showed a widespread, small‐nodular tumor, which exceeded the deepest border of the OCT image (Figure 1d).

**FIGURE 1:**
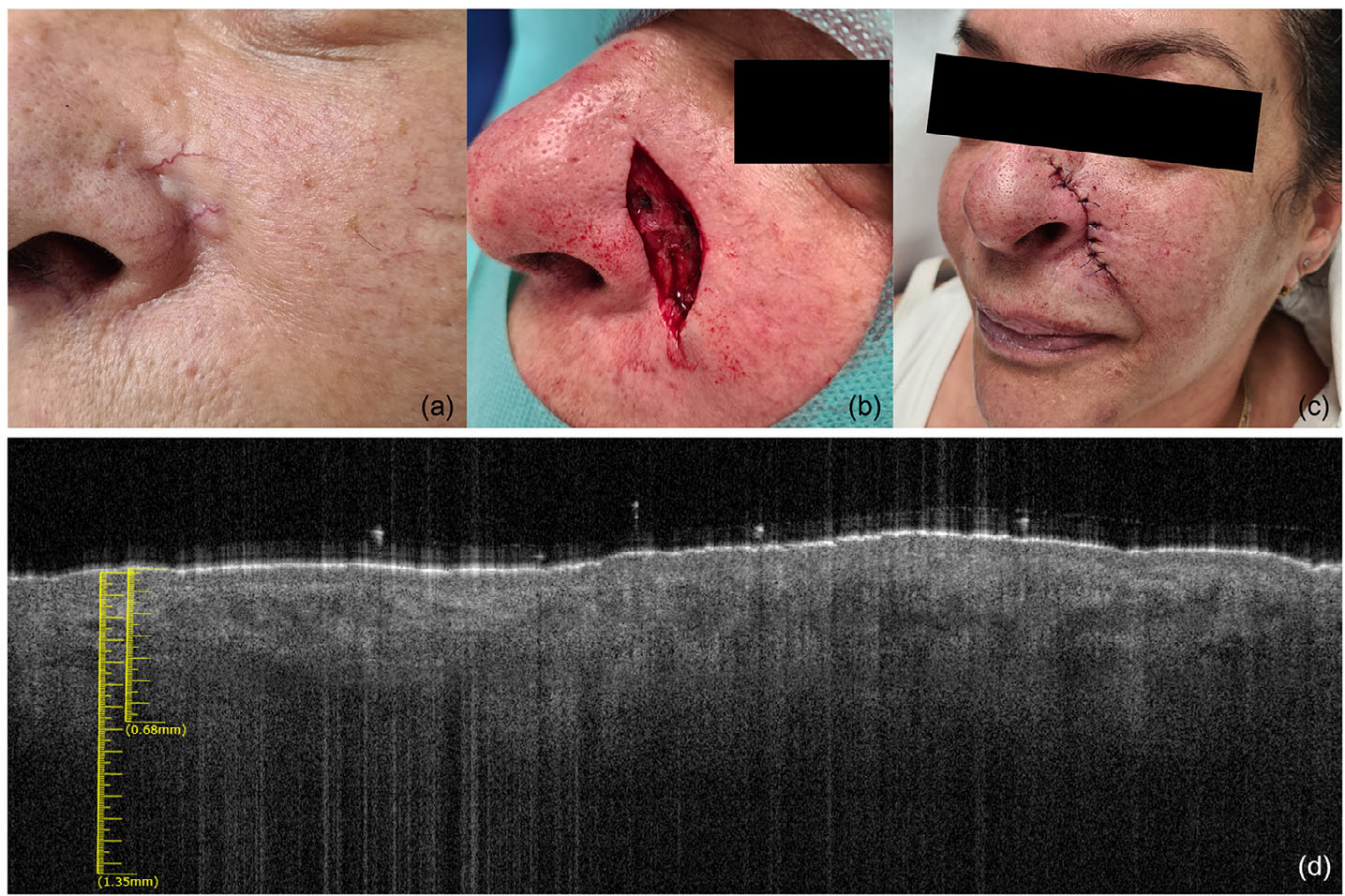
(a) Primary presentation of the patient with skin‐coloured plaque and prominent teleangiectasia; (b) Intraoperative; (c) Immediately postoperative; (d) OCT image showing an extensive, small‐nodular tumor. The basal border of the tumor is not visible on the OCT image.

A spindle biopsy showed a basaloid, small‐nodular tumor with abundant, dense, cell‐rich stroma, lacking epidermal connection (Figure 2a–c). There were mitotic figures and subtle peripheral palisading of the tumor cells. Inside some of the tumor nodules, round areas with a lighter cell population were visible (“ball‐like structures”, Figure 1c).^1^ Infiltrative growth of tumor nodules into superficial mimic muscle layers was visible (Figure 2b). Immunohistochemistry (IHC) showed strong BerEp4 and AE 1/3 positivity, markedly elevated proliferative activity in Ki‐67 stain, and, interestingly, enhanced density of CK20+ Merkel cells in some of the tumor nodules (Figure 2d–f).

**FIGURE 2:**
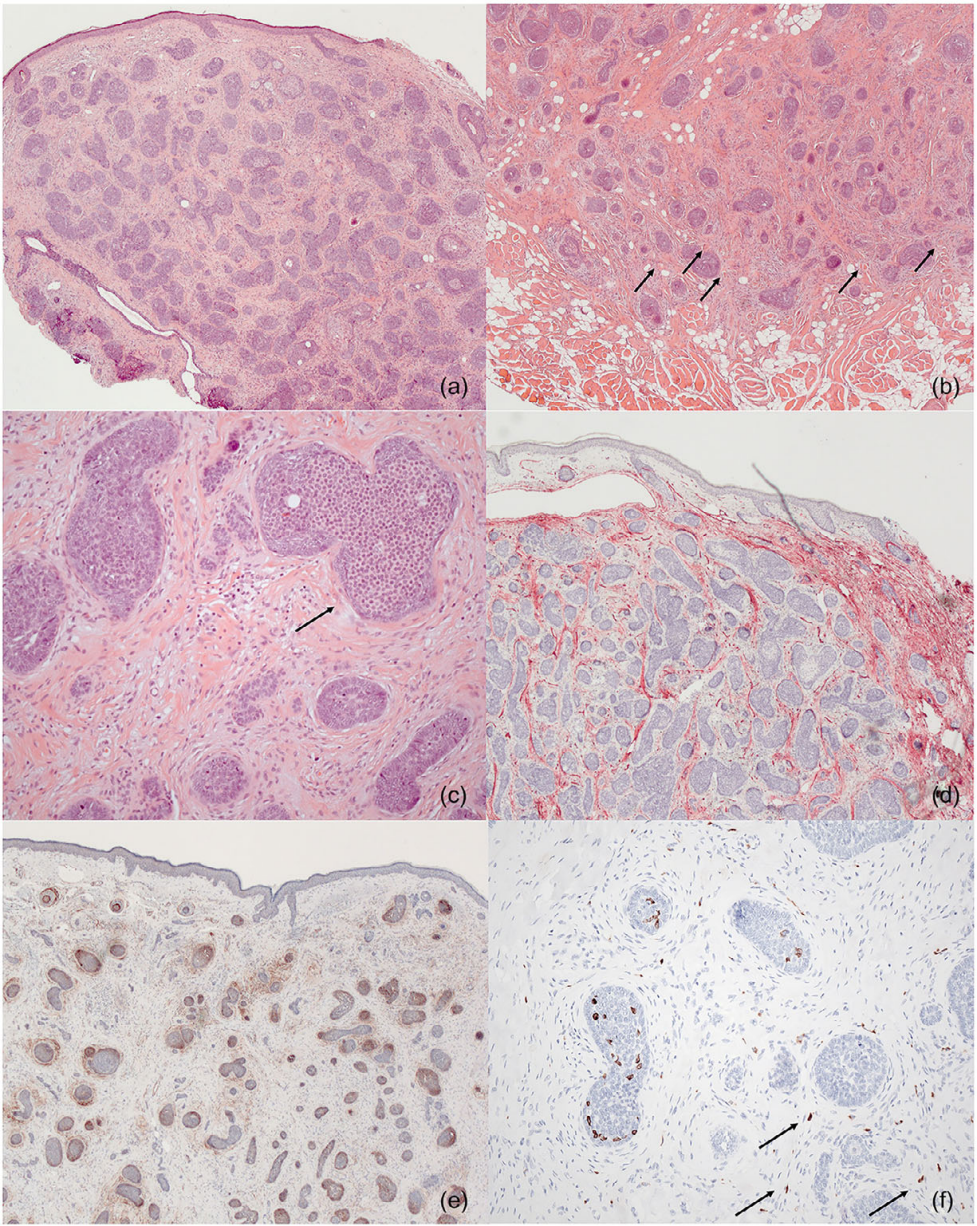
(a) H&E stain, x25 magnification: Small basaloid tumor nodules without epidermal connection and with extensive, cell‐rich tumor stroma. (b) H&E stain, x25 magnification: Infiltrative growth into mimic muscle (arrows) and subcutaneous fat tissue. (c) H&E stain, x100 magnification: Ball‐like structures (arrow). (d) CD34 stain, x25 magnification, red chromophore: Diffuse positive in tumor stroma and local connective tissue. (e) CD10 stain, x25 magnification, brown chromophore: Diffuse positive in tumor nodules and tumor stroma. (f) CK20 stain, x100 magnification, brown chromophore: single tumor nodules with increased CK20+ Merkel cells inside the nodules and in the peritumoral stroma (arrows), a typical finding for the perifollicular bulge region.

We diagnosed a plaque‐type trichoblastoma, a rare, locally infiltrative variant of trichoblastoma.

Clinical and histological differential diagnosis includes other follicular tumors like basal cell carcinoma (BCC) and “conventional” trichoblastoma (TB). These tumor entities are considered closely related, with differentiation towards the bulb‐papilla‐region, forming a “follicular tumor family”.^2^ While BCC is suspected to differentiate toward follicular bulb cells, TB differentiates toward both follicular bulb cells and follicular papilla cells.^2^ This explains why BCC only has sparse tumor stroma and retraction artifacts (clefts) located between tumor nodules and tumor stroma, while TB has abundant, cell‐rich tumor stroma and clefts located between the tumor stroma and the surrounding local connective tissue. Additionally, TB lacks an epidermal connection, which is often present in BCC, and it may show papillary mesenchymal bodies: Cell‐rich stromal nodules which resemble the follicular papilla and which are embraced by the tumor nodules in a claw‐like manner (not visible in our case). However, there are cases of TB with BCC‐like foci,^3^ and also reports of a trichoblastoma which was later revised to be a trichoblastic basal cell carcinoma.^4^, ^5^ These findings show that there is significant overlap between these entities.

Plaque‐type trichoblastoma shows some histological features that strongly resemble BCC, such as locally infiltrative growth (demonstrated by the muscular invasion in this case) and ill‐circumscribed, plaque‐like clinical appearance. At the same time, it shares features with TB (e. g. abundant, cell‐rich stroma). According to the literature, its behavior is considered benign or low‐grade malignant with local infiltration, but without reports of distant metastasis.^4^, ^6^, ^7^, ^8^ Likewise, our patient had to undergo two excisions to achieve tumor‐free margins.

Histological diagnosis is based mostly on HE‐morphological examination (Table 1).

**TABLE 1:** Comparison of BCC, Plaque‐type Trichoblastoma and Trichoblastoma.

		BCC	Plaque‐type trichoblastoma	Trichoblastoma
**H&E findings**	**Architecture**	Ill defined	Ill defined	Well defined
	**Small basophile tumor nodules**	Yes	Yes	Yes
	**Peripheral palisading of tumor cells**	Yes	Yes	Yes
	**Tumor stroma**	Sparse	Cell‐rich, extensive	Cell‐rich, moderate
	**Invasive growth**	Yes	Yes	No
	**Tumor is easy to pop out of the skin**	No	No	Yes
	**Clefts**	Between tumor nodules and tumor stroma	No ^10^	Between tumor stroma and surrounding connective tissue
	**Connection between epidermis and tumor**	Yes	No	No ^7^
	**Ball‐like structures**	No	Yes (visible in our case) No further data available	No
	**Papillary mesenchymal bodies**	No	Yes	Yes

Although many different IHC stainings are suggested to aid in the distinction of these entities, most of them have limited discriminative power (online supplementary Table S1). CK20 expression seems to be the most helpful IHC analysis in the distinction of BCC and “conventional” TB: While TB may show elevated numbers of CK20+ Merkel cells inside the tumor nodules, BCCs do not.^9^ To our knowledge, there is no information regarding CK20 expression in plaque‐type trichoblastoma apart from this case, showing a focally increased number CK20‐positive cells in some of the tumor nodules.

In some cases, plaque‐type trichoblastomas were shown to harbor somatic mutations in the *PTCH1* gene, a tumor suppressor gene and part of the sonic hedgehog pathway. *PTCH1* mutations are a characteristic finding of BCC and Goltz‐Gorlin Syndrome.^6^, ^10^ However, we could exclude a mutation in the *PTCH1* gene in our case.

In conclusion, plaque‐type trichoblastoma is a rare, locally infiltrative tumor with differentiation towards the follicular bulb and papilla, and which forms a “tumor family” together with BCC and “conventional” TB. A distinction of these tumors is clinically and histologically challenging due to their close relatedness. Our case demonstrates that plaque‐type trichoblastoma also shows focally increased CK20+ Merkel cells, like “conventional” TB. Due to the importance of architectural and morphological factors for diagnosis, excisional biopsies should be strongly preferred over shaving biopsies. We do not feel that OCT offers a relevant advantage due to its limited penetration depth compared to the tumor depth. We recommend complete excision due to the locally infiltrative growth pattern.

## CONFLICT OF INTEREST STATEMENT

None.

## Supporting information



Supporting Information
