# Supplementary material for: Plaque‐type trichoblastoma and its siblings: A (follicular tumour) family history
Source: J Dtsch Dermatol Ges. 2026 Mar 19;24(11):1643–6. doi: 10.1111/ddg.70255 (PMC13749585; doi:10.1111/ddg.70255)
Supplement: Supplementary file 1 [file DDG-24-1643-s001.docx]

Online Supplementary table

**Supplementary Table S1:**

Immunohistochemical comparison of BCC, Plaque-type Trichoblastoma and Trichoblastoma

|  |  | **BCC** | **Plaque-type trichoblastoma** | **Trichoblastoma** |
| --- | --- | --- | --- | --- |
| **Immuno-histo-chemical findings** | **BerEP4** | Positive in tumor nodules  ^7^ | Positive in tumor nodules  ^4^  Negative or patchy positive  ^7^ | Positive in tumor nodules  ^7^ |
|  | **CK20** | No positive cells in tumor nodules  ^9^ | Focally increased CK20+ merkel cells inside tumor lobules (visible in our case)  No further data available | Increased positive cells in tumor nodules  ^9^ |
|  | **CD10** | Positive in tumor nodules, negative in tumor stroma  ^7^ | Diffuse positive in tumor stroma and local stroma  ^10^ and ^7^ | Positive in tumor stroma  ^7^ |
|  | **CD34** | Negative in tumor nodules and in tumor stroma  ^7^  Positive in tumor stroma  ^4^ | Diffuse positive in tumor stroma and surrounding stroma  ^7^  Positive in tumor stroma  ^4^^,^^10^ | Positive in tumor stroma  ^7^ |
|  | **Bcl-2** | Diffuse positive  ^7^ and ^4^ | Positive in direct tumor stroma  ^7^  Diffuse positive in tumor nodules  ^4^ | Positive in peripheral tumor nodules  ^7^ and ^4^ |
|  | **PHLDA1** | Negative  ^8^ | Variable degree  ^8^ | Positive ^11^ |

Orange stained fields= fields in which the findings of different authors do not match

Tumor stroma = stroma which comprises part of the tumor, directly adjacent to- and surrounding the tumor nodules

Local stroma = non-tumorous, healthy stroma, into which tumor nodules and tumor stroma are embedded
